# Escalation of care planning on an older adult inpatient unit during the COVID-19 pandemic

**DOI:** 10.1192/bjo.2021.275

**Published:** 2021-06-18

**Authors:** Alexander McDermott, Jennifer Rankin

**Affiliations:** Angelton Clinic – Cwm Taf Morgannwg University Healthboard

## Abstract

**Aims:**

Initial planning during the first wave of the COVID-19 pandemic involved difficult decision making for many clinicians. The Older Adult Mental Health Wards in Bridgend were relocated from the district general hospital (Princess of Wales) and merged at Angelton Clinic, an off site separate unit. It was therefore essential that patients had clear escalation of care plans as access to medical input was limited and transfer to hospital potentially not appropriate in the later stages of chronic illness such as dementia.

The initial aim of the PDSA cycle was to assess the level of compliance with Do Not Attempt Resuscitation (DNAR) discussions and if appropriate, DNAR documentation. The other aim was to assess the utilisation of Escalation of Care plans.

**Method:**

An audit of patients MDT medical notes on 38 admitted to Angelton clinic was carrired out in March. It was documented if the patient had a clear DNAR or Escalation plan that was easily accessible in the front of the notes. The guidelines compared to were the GMC recommendations that patients 12 months of should have a discussion about risks and benefits associated with Cardiopulmonary Resuscitation. If the patient lacks capacity a best interest decision should be made with nearest relatives. Discussions should also be had with patients and family in in regards to and transfer to a medical ward.

Upon completion of the initial PDSA cycle, views were sought from the wider MDT a new escalation of care proforma was designed. This was implemented by education and communication with members of the medical team. This was to be clearly placed in the notes, with the DNAR form if that was appropriate.

**Result:**

All inpatient notes were audited at Angelton Clinic in March 2020. It was found that only 18% of patients had Escalation of Care plans in comparison to 84% of notes which had DNAR forms. Previous escalation of care forms were not being utilised appropriately.

Upon implementation of the Escalation of Care proforma, a re-audit of the audit cyle was completed. In July 2020 it was found that 78% of notes had completed Escalation of Care forms with 83% had completed DNAR forms.

**Conclusion:**

To enable ongoing sustained improvement, the unit Nurse Practitioner will champion its completion. The audit findings have been shared with the newly rotated junior doctors and proformas were made available on all inpatient wards.

